# In Vitro Antifungal Activity of Amphotericin B-Encapsulated Silk Fibroin-Chitosan Nanoparticles Against *Fusarium solani* Isolates from Keratitis Patients

**DOI:** 10.3390/pharmaceutics17091170

**Published:** 2025-09-08

**Authors:** Rossukon Khotcharrat, Sangly P. Srinivas, Yordhathai Thongsri, Wanachat Thongsuk

**Affiliations:** 1 Department of Ophthalmology, Faculty of Medicine, Naresuan University, Phitsanulok 65000, Thailand; 2 School of Optometry, Indiana University, Bloomington, IN 47405, USA; srinivas@indiana.edu; 3 Cellular and Molecular Immunology Research Unit, Faculty of Allied Health Sciences, Naresuan University, Phitsanulok 65000, Thailand; yordhathait@nu.ac.th; 4 Department of Medical Technology, Faculty of Allied Health Sciences, Naresuan University, Phitsanulok 65000, Thailand; 5 Department of Optometry, Faculty of Allied Health Sciences, Naresuan University, Phitsanulok 65000, Thailand

**Keywords:** silk fibroin nanoparticles, amphotericin B, fungal keratitis, *Fusarium solani*, ocular drug delivery, chitosan, mucoadhesion, antifungal therapy

## Abstract

**Background:** Fungal keratitis is a serious ophthalmic problem due to low antifungal medication penetration and bioavailability at the ocular surface, necessitating novel delivery strategies for successful therapeutic outcomes. This study created amphotericin B-loaded silk fibroin nanoparticles (AmB-SFNs) as a targeted drug delivery platform for long-term ocular antifungal therapy. **Methods**: Silk fibroin-chitosan nanoparticles were produced using a precipitation technique, with chitosan coating for mucoadhesion and polyethylene glycol-400 surface stability. Clinical fungal isolates from keratitis patients were identified as species by morphological and molecular analysis, followed by in vitro antifungal susceptibility testing. **Results**: The optimized formulation produced spherical AmB-SFNs with an average diameter of 220 nm, a positive zeta potential of +34 mV, and a maximum amphotericin B entrapment effectiveness of 76%. Molecular identification confirmed that all five clinical isolates were *Fusarium solani*. AmB-SFNs showed strong antifungal activity against all tested isolates, with a minimum inhibitory dose of 50 μg/mL (0.25% *w*/*v*). **Conclusions**: The developed nanoparticulate system has optimal characteristics for enhanced corneal drug delivery, such as appropriate particle size for tissue penetration and mucoadhesive properties for prolonged ocular residence time, suggesting that this nanoparticulate system warrants further investigation in vivo to evaluate its potential for clinical translation in treating Fusarium keratitis and as a platform for topical ophthalmic therapies.

## 1. Introduction

Fungal keratitis (FK), caused primarily by Candida species, is the leading cause of vision loss worldwide [1]. It is a severe corneal disease that can result in permanent vision loss and must be treated as early as possible [1]. Topical Amphotericin-B (AmpB) (0.1–0.3%) is the standard treatment for Candida and related fungi ocular infections [2]. The current formulation of AmpB eye drops contains deoxycholate, which is required to solubilize the highly hydrophobic AmpB, making instillation painful and leading to poor compliance and worsening of symptoms [3]. Hence, therapeutic management of AmpB eye drops remains difficult.

Although topical ocular administration of eye drops is a common method for treating the ocular surface, the drug bioavailability after eye instillations is very low. This limitation is due in part to the fact that tear film renewal, blinking reflex and nasopharyngeal drainage reduces the amount of time drug-containing eye drops are exposed to the ocular surface, resulting in low drug bioavailability [4,5,6].

As a result, in order to be effective in treatment, eye drops must be used frequently and in high concentrations. This has both local and systemic implications. Furthermore, it has the potential to cause side effects such as eye irritants and corneal abrasion, which reduces patient compliance. If the symptoms are so severe that eye drops are no longer effective, additional procedures, such as injections into the anterior chamber of the eye, injections into the cornea, and injections under the conjunctiva, are required. Other, much more expensive drugs, such as natamycin and voriconazole, may be considered by an ophthalmologist [7]. If the infection spreads, the pus in the anterior intraocular cavity must be removed. When the cornea has been penetrated or the infection cannot be controlled, corneal transplant surgery from a donor eye is performed. If the infection has spread beyond the cornea, eye surgery may be required to prevent further invasion of the bloodstream and brain, which could be fatal [8].

To overcome the highly hydrophobic AmpB, whose use is restricted due to its toxicity and poor solubility, and to improve its bioavailability on the ocular surface, nanoparticles have emerged as a novel platform for increasing topical ocular bioavailability. Because of their small particle size, they may increase corneal residence time [4]. As a result, this could improve drug therapeutic efficacy while reducing undesirable drug side effects. Furthermore, because the drug is incorporated into nanoparticles, it may reduce ocular irritation [4,9]. Despite the fact that nanoparticles can be made from a variety of materials, biodegradable polymers and mucous membranes are the most commonly used as ophthalmic materials [10,11].

Silk fibroin (SF) is a structural protein derived from the *Bombyx mori* silkworm that has gained considerable attention as a nanocarrier material. Its intrinsic advantages including biocompatibility, biodegradability, and low toxicity make it highly suitable for biomedical use [10,12]. Approved by the FDA, SF has already been translated into clinical applications ranging from surgical sutures and tissue engineering scaffolds to surface coatings and advanced drug delivery platforms [10]. Chitosan is another widely used polymer as an ocular drug particulate carrier because its positively charged structure can interact with the mucus layer at the ocular surface via ionic interaction [4,6].

Despite the promise of both polymers individually, their combination as a dual-polymer system for ocular antifungal therapy has not been reported to date. In this study, we propose a novel formulation in which AmpB is encapsulated in silk fibroin nanoparticles and further coated with chitosan. This synergistic design integrates the drug-stabilizing capacity of silk fibroin with the mucoadhesive and permeability-enhancing properties of chitosan, resulting in improved ocular retention, stability, and antifungal efficacy. To the best of our knowledge, this is the first report of a silk fibroin–chitosan dual-polymer carrier applied to ocular antifungal therapy, specifically targeting *Fusarium solani*, a clinically challenging pathogen in FK.

Consequently, the goal of this study was to develop an ophthalmic formulation of Amphotericin B-loaded silk fibroin nanoparticles coated with chitosan (AmB-SFNs) to overcome the current limitations of ocular antifungal therapy. This dual-polymer nanoparticle system, which uniquely combines the stabilizing properties of silk fibroin with the mucoadhesive and permeability-enhancing features of chitosan, offers a novel and promising strategy to enhance drug stability, ocular retention, and antifungal efficacy, particularly against *Fusarium solani.*

## 2. Materials and Methods

### 2.1. Materials

Amphotericin B (AmB) powder was purchased from Sigma-Aldrich (St. Louis, MO, USA). Degummed silk yarns derived from *Bombyx mori* were obtained from Bodin Thai Silk Khorat Co., Ltd. (Nakhon Ratchasima, Thailand). Chitosan from shrimp shells (CS; MW 30 kDa, 95% degree of deacetylation) was supplied by Aquapremier, Inc. (Bangkok, Thailand). Polyethylene glycol 400 (PEG400) was obtained from Nam Siang Co., Ltd. (Bangkok, Thailand). All solvents and reagents were of analytical grade and procured from RCI Labscan (Bangkok, Thailand).

### 2.2. Silk Fibroin Extraction

Silk fibroin (SF) was prepared following a modified protocol described previously [4]. In brief, 5 g of degummed silk yarns were cut into short fibers and dissolved in a mixture of CaCl_2_:H_2_O:Ca(NO_3_)_2_:EtOH at a weight ratio of 30:45:5:20. The mixture was subjected to microwave heating at 900 W for 2 min until a clear solution was obtained. The resulting SF solution was dialyzed in a snakeskin pleated dialysis membrane (MWCO 10,000) against distilled water at room temperature for 3–5 days to remove residual salts. The dialyzed solution was then centrifuged at 10,000 rpm and 4 °C for 30 min to eliminate impurities and silk aggregates formed during dialysis. Finally, the clarified SF solution was lyophilized using a freeze dryer (Heto PowerDry LL3000, Thermo Fisher, Walthamm, MA, USA) at 1.9 × 10^−4^ Torr and −55 °C, and the resulting powder was stored in sealed plastic bags at −80 °C until used.

### 2.3. Preparation of AmB-Loaded Fibroin Nanoparticles Coated with Chitosan

Amphotericin B-loaded silk fibroin nanoparticles (AmB-SFNs) were prepared using a two-step process involving nanoparticle formation by precipitation, followed by chitosan (CS) coating and PEG400 stabilization.

For nanoparticle synthesis, SF solution (1% *w*/*v* in deionized water) was added dropwise to ethanol at a 1:1 ratio (SF:EtOH) under continuous stirring (~750 rpm). The mixture was stirred at room temperature for 30 min to allow spontaneous formation of fibroin nanoparticles. The resulting nanoparticles were stored at 4 °C for 24 h. The nanoparticles were then washed twice with sterile water by centrifugation at 18,000 rpm for 30 min (J2-MC, Beckman Coulter, Inc., Brea, CA, USA) using a 20 µL glycerol cushion at the bottom of the tube to prevent aggregation. The washed nanoparticles were re-dispersed in deionized water, followed by vortexing and sonication twice at 40% amplitude for 30 s to achieve uniform dispersion.

To impart mucoadhesive properties, the nanoparticles were coated with CS by adding a 1% (*w*/*v*) CS solution in 1% (*v*/*v*) acetic acid, followed by gentle stirring at ~500 rpm for 20 min at room temperature. Subsequently, PEG400 was added dropwise under continuous stirring (~500 rpm) for an additional 20 min to enhance colloidal stability. The final nanoparticles were washed twice with deionized water using the same centrifugation method as described above.

In order to load amphotericin-B (AmB) into the nanoparticles, various amounts of AmB were evaluated. The selected amount of AmB powder was dissolved in the SF solution and allowed to equilibrate for 2 h prior to the addition of ethanol. The resulting nanoparticles were then coated with CS and stabilized with PEG400 following the procedures described above. All experiments were performed in triplicate.

### 2.4. Physicochemical Characterization of AmB-SFNs

#### 2.4.1. Morphology

The morphology of the AmB-SFNs was characterized using scanning electron microscopy (SEM; 1455VP, LEO Electron Microscopy Ltd., Cambridge, UK). The SFNs suspension was placed on a glass cover slip (to provide an ultra-flat, clean surface) mounted on an SEM stub and allowed to air-dry at room temperature. The dried SFN samples were sputter-coated with a ~10 nm layer of gold to render them conductive and minimize charging artifacts during electron beam imaging. The samples were then examined at a magnification of 10,000×.

#### 2.4.2. Particle Size and Surface Charge

The mean particle size and size distribution of AmB-SFNs was measured by dynamic light scattering (DLS) using ZetaPAL/90plus (Brookhaven Instrument, Holtsville, NY, USA), equipped with a 35 mW HeNe laser diode (632.8 nm; JDS Uniphase, San Jose, CA, USA) and a BI-200SM goniometer with an EMI-9863 photomultiplier tube connected to a BI-9000AT digital correlator (Brookhaven Instrument, Holtsville, NY, USA). Aliquots of AmB-SFNs were diluted in deionized water, and particle size measurements were performed at 25 °C at a 90° detection angle with 10 repeated measurements per sample. The raw data were analyzed using cumulative analysis to obtain the average particle size.

Surface charge was measured as zeta potential using phase analysis light scattering on the ZetaPAL/90plus instrument (Brookhaven Instrument, Holtsville, NY, USA). Samples were prepared by re-dispersing SFNs in DI water [4], and zeta potential values were calculated from electrophoretic mobility using the Smoluchowski approximation.

#### 2.4.3. Drug Entrapment Efficiency

The entrapment efficiency (EE) of AmB-SFNs was assessed via an indirect method [10]. In this procedure, 1 mL of each formulation was subjected to centrifugation at 14,000 rpm for 30 min, and the supernatant was collected to determine the amount of unentraped AmB by UV–Vis spectrophotometer at 405 nm.

### 2.5. Clinical Isolates

A total of 5 clinical isolates were obtained from the central laboratory of Naresuan University Hospital (Phitsanulok, Thailand), where they had been originally isolated from keratitis patients infected with filamentous fungi. Briefly, each of the five clinical fungal isolates was subcultured from sabouraud dextrose agar (SDA) slant stocks onto fresh SDA plates by streaking, followed by incubation at 35 °C for 48 h. Single colonies were isolated using the cross-streak method, then subcultured and maintained as SDA slant stocks at 4 °C for subsequent studies.

### 2.6. Fungal Morphology

Microscopic characteristics of isolates were studied using light microscopy. The clinical isolates sample were freshly cultured aerobically on SDA plate by cross-streak method at 35 °C for 48 h. As fungal growth was observed, a lactophenol-cotton blue solution wet mount was prepared for microscopic examination of fungi. Briefly, the slide was stained before being covered in mycelia, which was subsequently covered with slips. Following that, the slide was viewed using a microscope. Moreover, the cultural characteristics were also studied.

### 2.7. Molecular Characterization

The purified fungal colonies were sub-cultured, and the fungal genomic DNA was extracted and purified using the DNeasy Plant Mini Kit (Qiagen, Hilden, Germany) following the directions from the manufacturer. DNA samples were stored at –20 °C. 18S rRNA gene sequences and their associated transcribed spacers (internal transcribed spacer; ITS) were performed by the DNA Sequencing Service of Macrogen (Seoul, Republic of Korea). Following sequencing, DNA sequences were analyzed using Chromas software version 2.3 and compared against the NCBI fungal database using the Basic Local Alignment Search Tool (BLASTn, v.2.6.0).

### 2.8. Antifungal Susceptibility

Antifungal susceptibility test was performed to evaluate the potent antifungal activity of AmB-SFNs against clinical isolates. It was performed as described in the Clinical and Laboratory Standards Institute (CLSI) M51-A with modifications [13]. Briefly, the single-cell isolation from cross streak method was freshly cultured aerobically on SDA plate at 35 °C for 48 h (or until they reached full growth). Use a sterile cork borer (6 mm) to drill into the colony’s center of the cultured isolate and lay it down on a fresh SDA plate. After that, 20 μL of AmB-SFNs, containing 50, 100, 150, or 200 µg of AmB per disc, were applied to sterile discs, with AmB deoxycholate solution at corresponding concentrations used as the control. All discs were air-dried at room temperature, then placed on agar plates and incubated aerobically at 35 °C for 48 h. The diameters of inhibition zones were subsequently measured and recorded in millimeters.

### 2.9. Statistical Analysis

All measurements were carried out in triplicate, with results expressed as mean ± standard deviation. Statistical analyses included paired *t*-tests, one-way ANOVA with post hoc tests, and two-way factorial ANOVA. For the two-way ANOVA, Tukey’s HSD was applied for multiple comparisons to evaluate effects specific to isolates and concentrations. A *p*-value ≤ 0.05 was considered statistically significant.

## 3. Results and Discussion

### 3.1. Physicochemical Properties

**Figure 1:**
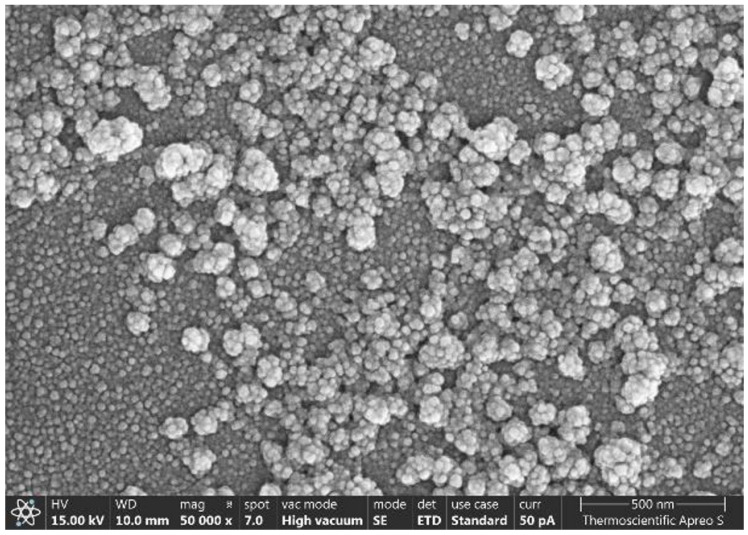
SEM micrographs showing the spherical morphology of AmB-SFNs. Scale bar: 500 nm.

Morphological analysis by SEM demonstrated that AmB-SFNs possessed uniform spherical geometry with smooth surface characteristics and homogeneous particle distribution (Figure 1), suggesting that the precipitation methodology integrated with chitosan surface modification and PEG400 stabilization yielded consistent processing parameters with negligible influence on nanoparticle morphology [4]. As shown in Table 1, the formulations exhibited superior uniformity, as evidenced by polydispersity index values consistently below 0.2, indicating narrow particle size distribution essential for reproducible pharmaceutical performance [4,6]. Under optimal formulation conditions, AmB-SFNs demonstrated mean particle diameters ranging from 263–333 nm, positioning them within a size range (100–1000 nm) that is theoretically favorable for ocular drug delivery systems and has the potential to facilitate corneal permeation while minimizing premature elimination via nasolacrimal drainage [4,5,6]. The pronounced positive surface charge (>+30 mV), conferred predominantly by chitosan functionalization, is expected to promote electrostatic interactions with negatively charged ocular mucins, thereby extending precorneal residence time and enhancing drug bioavailability [4,5,6]. Concurrently, the high zeta potential contributes to colloidal stability by minimizing interparticle aggregation through electrostatic repulsion mechanisms [5]. It should be noted, however, that the present study did not investigate the long-term physicochemical stability of AmB-SFNs. Comprehensive stability assessments under diverse storage conditions remain an essential prerequisite for clinical translation and will be addressed in future investigations.

### 3.2. Entrapment Efficacy and Dissolution Profile of Amphotericin B

Beyond the effects on particle size and surface charge, the initial AmB concentration also significantly influenced drug entrapment efficiency (Table 1). The initial concentration of AmB represents a critical formulation parameter that significantly influences the physicochemical properties and drug-loading capacity of AmB-SFNs. In this investigation, optimal entrapment efficiency of 74.45% was achieved at an AmB concentration of 0.16 mg/mL. Concentrations exceeding this threshold resulted in diminished entrapment efficiency, attributed to matrix saturation and potential drug precipitation within the nanoparticle core. This observation is consistent with established principles in nanoparticle drug delivery systems, where excessive drug loading compromises encapsulation efficiency through destabilization of particle formation or induction of drug aggregation phenomena [4,5,6,9].

Concomitantly, elevated AmB concentrations demonstrated a proportional increase in particle size, presumably resulting from enhanced drug incorporation into the nanoparticle matrix, consequently yielding larger particle dimensions. The positive surface charge (zeta potential) exhibited concentration-dependent enhancement, which confers advantageous mucoadhesive properties through electrostatic interactions with the anionic ocular surface, thereby prolonging ocular residence time [4,6]. Nevertheless, excessively high surface charge values may compromise nanoparticle colloidal stability and adversely affect drug entrapment efficiency, as evidenced in formulations containing the highest AmB concentration [14,15].

These experimental findings corroborate existing literature on silk fibroin nanoparticles for ophthalmic applications, emphasizing that judicious optimization of drug concentration is paramount for maintaining nanoparticle stability, appropriate size distribution, and maximal drug loading capacity, ultimately aiming to facilitate effective ocular drug delivery with enhanced retention and sustained release characteristics [10]. Based on these in vitro properties, studies suggest that mucoadhesive systems like AmB-SFNs have the potential to exhibit enhanced ocular retention and improved safety profiles compared to conventional formulations [9,11]. Therefore, controlling the initial AmB concentration, particularly at the optimal level of 0.16 mg/mL in this formulation system, helps achieve higher drug entrapment efficiency while maintaining nanoparticle size and surface properties suitable for ocular delivery.

In addition, the integration of nanoparticle formulations into gel-based ocular delivery systems has emerged as a promising strategy to further prolong precorneal residence time and ensure sustained release at the site of infection. Hydrogels not only provide mucoadhesive characteristics that enhance contact with the ocular surface but also protect entrapped nanoparticles against premature clearance by tear fluid [16,17]. Previous studies have demonstrated that embedding antifungal or antibacterial nanoparticles within in situ gelling systems leads to improved bioavailability, reduced dosing frequency, and better therapeutic outcomes compared to eye drops alone [18,19,20,21]. Thus, incorporating AmB-SFNs into gel-forming platforms could represent a synergistic approach, combining the benefits of nanoparticle encapsulation with the sustained release profile of gels, thereby aligning with advanced strategies in ocular drug delivery.

### 3.3. Morphological Identification of Fungal Isolates

**Figure 2:**
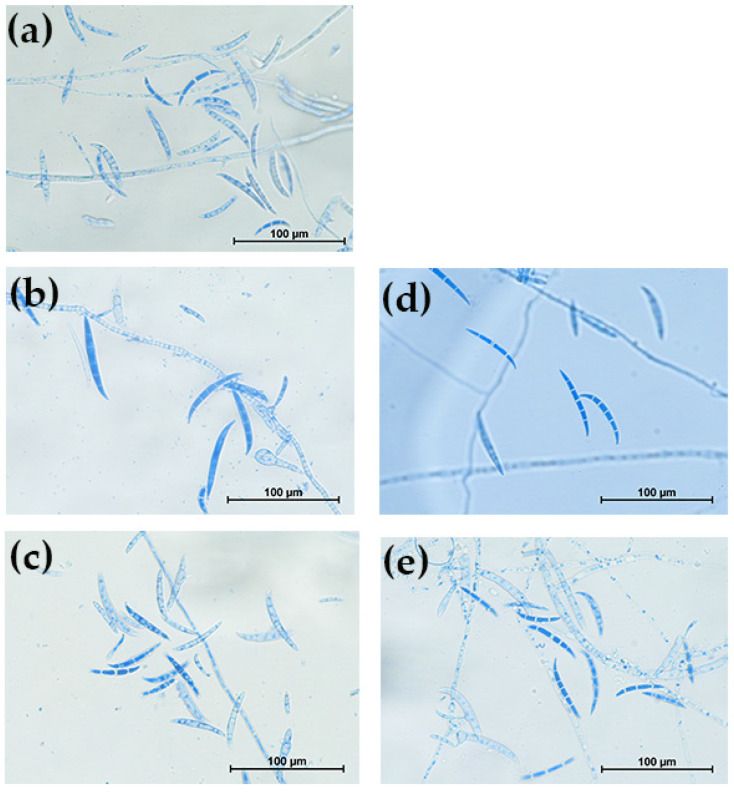
Microscopic images of lactophenol cotton blue-stained fungal isolates. (**a**): Clinical isolate 1 (E1). (**b**): Clinical isolate 2 (E2). (**c**): Clinical isolate 3 (E3). (**d**): Clinical isolate 4 (E4). (**e**): Clinical isolate 5 (E5). Scale bar: 100 µm.

**Figure 3:**
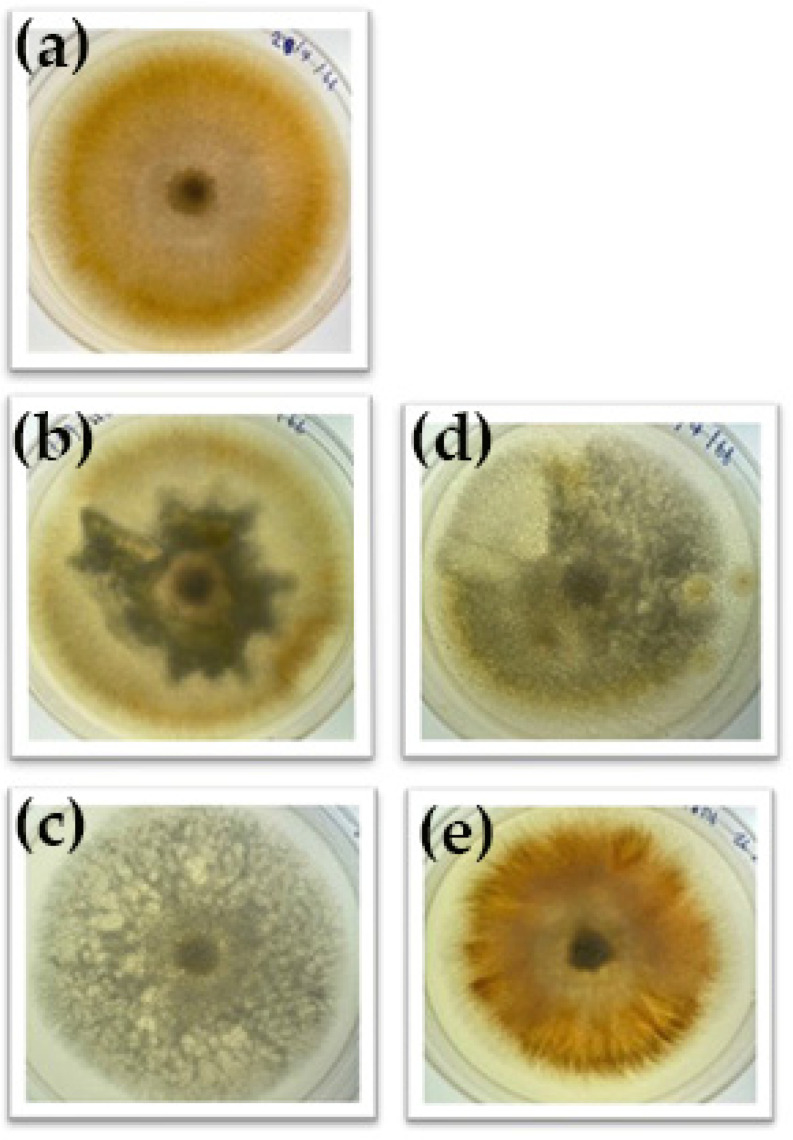
Purified fungal colonies were grown on 90 mm sabouraud dextrose agar (SDA) plates shows typically flat, spreading, wooly colony growths, initially appearing white or grey-white in color, then producing a variety of colors when grown in culture. (**a**): Clinical isolate 1 (E1). (**b**): Clinical isolate 2 (E2). (**c**): Clinical isolate 3 (E3). (**d**): Clinical isolate 4 (E4). (**e**): Clinical isolate 5 (E5).

All five fungal isolates collected from keratitis infections were identified using the typical colony morphology on SDA and microscopic characteristics following lactophenol cotton blue staining. As demonstrated in Figure 2, all isolates displayed long chains of conidia and septate mycelia, which are key identifying features of *Fusarium* spp. While colonies on SDA produced flat, spreading, wooly colony growths that appeared white or grey-white at first, they eventually turned a variety of colors when cultured, as shown in Figure 3. These colony traits align well with the typical morphology described for Fusarium species [22,23].

The morphological characteristics strongly suggested that the isolates belonged to the Fusarium genus, a significant causative agent of fungal keratitis worldwide, particularly in tropical regions [23]. However, given the morphological similarities among Fusarium species complexes, molecular analysis was additionally performed to enable accurate species-level identification in this study.

### 3.4. Molecular Characterization and Phylogenetic Analysis

Clinically, species-level identification of unknown *Fusarium* isolates is important because different species exhibit variable susceptibility to antifungal agents, which directly impacts treatment selection. Molecular genetic analysis plays a critical role in resolving monophyletic *Fusarium* species and provides a more accurate basis for guiding appropriate antifungal therapy.

DNA sequencing of partial gene regions was employed to complement morphological identification of *Fusarium* species. The nuclear ribosomal internal transcribed spacer (ITS) sequence should be sufficient to identify most isolates as belonging to the correct complex or *Fusarium* genus, but it will not be sensitive enough to distinguish between species within the complex.

**Figure 4:**
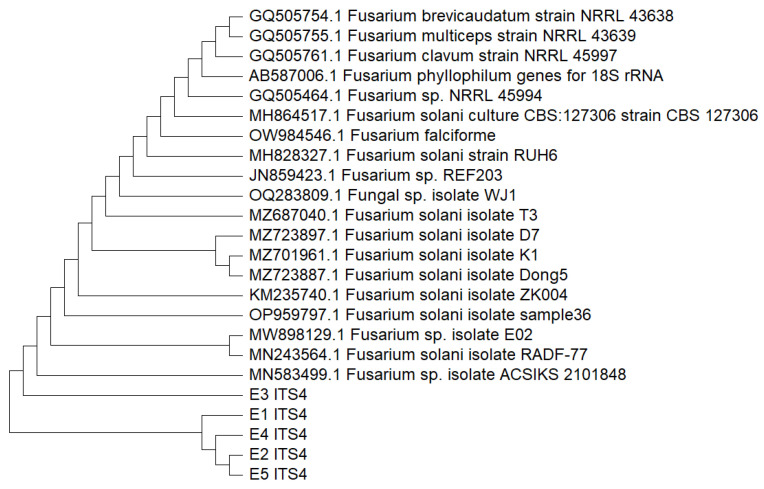
Phylogenetic tree of all 5 fungal isolated were closely related and identified as *Fusarium solani*.

After comparing the nucleotide sequences with the NCBI GenBank database and identifying similar traits using the BLAST + 2.16.0 tool, it was discovered that the identity of all five isolates exhibited molecular characteristics that matched to *Fusarium solani* (99% similarity), A phylogenetic tree was created by the neighbor tree joining method as shown in Figure 4.

### 3.5. Antifungal Efficacy

**Figure 5:**
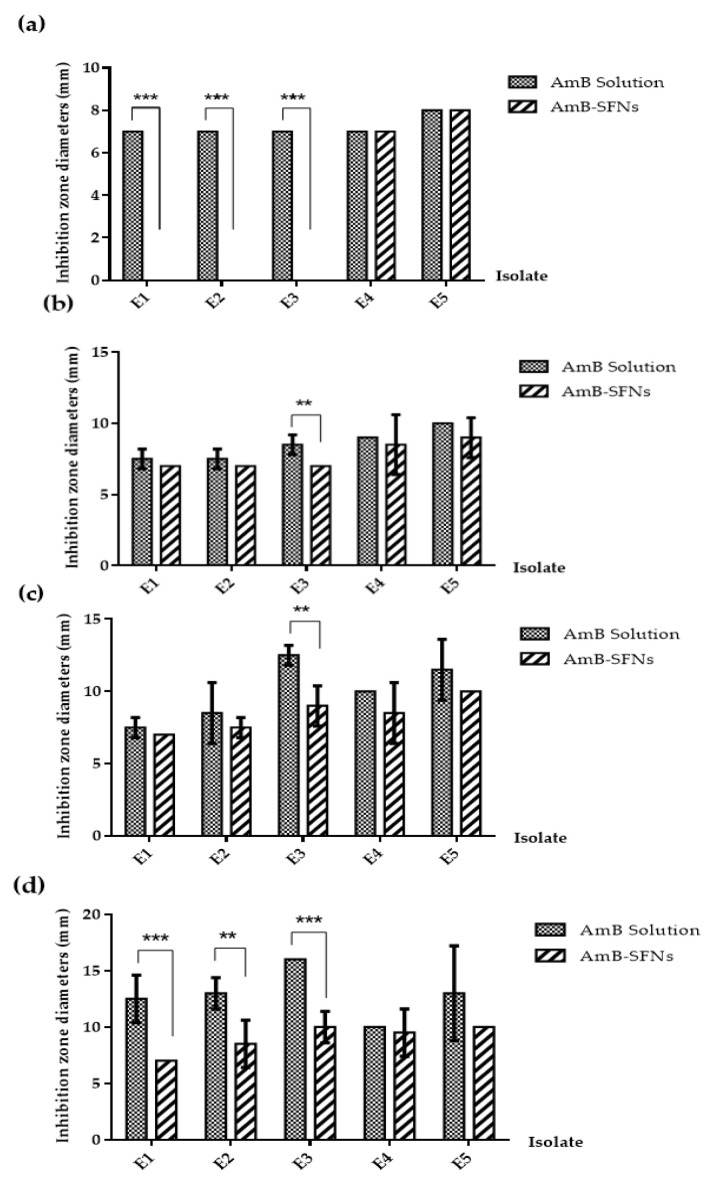
Antifungal activity comparison between AmB Solution and AmB-SFNs against different fungal isolates. (**a**) 50 μg AmB concentration—inhibition zone diameter (mm) showing highly significant differences (*** *p* ≤ 0.001) for isolates E1, E2, and E3, with AmB Solution demonstrating superior activity. (**b**) 100 μg AmB concentration—inhibition zone diameter (mm) showing significant difference (** *p* ≤ 0.01) for isolate E3 only. (**c**) 150 μg AmB concentration—inhibition zone diameter (mm) showing significant difference (** *p* ≤ 0.01) for isolate E3 only. (**d**) 200 μg AmB concentration—inhibition zone diameter (mm) showing highly significant differences for isolates E1 (*** *p* ≤ 0.001), E2 (** *p* ≤ 0.01), and E4 (*** *p* ≤ 0.001). Data represent mean ± standard error. AmB Solution (solid gray bars) and AmB-SFNs (striped bars) were tested against five different fungal isolates (E1–E5). Statistical significance: *** *p* ≤ 0.001 (extremely significant), ** *p* ≤ 0.01 (highly significant).

**Figure 6:**
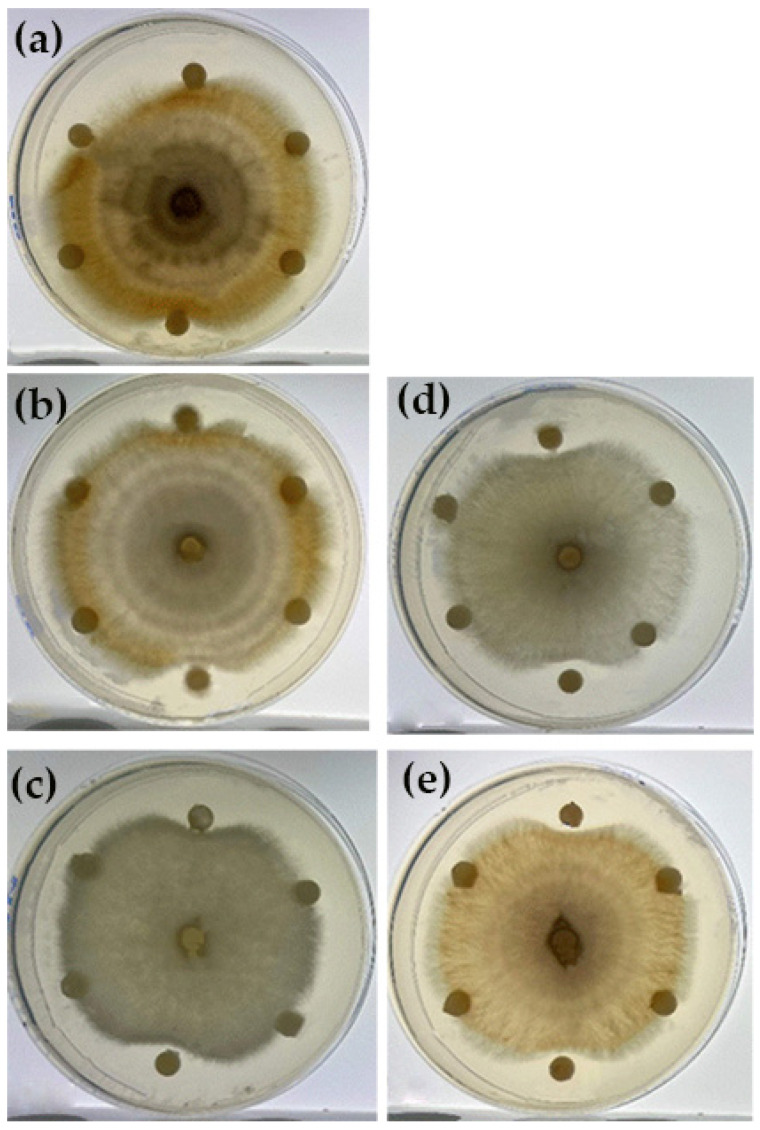
Antifungal susceptibility testing using disc diffusion method. (**a**): Clinical isolate 1 (E1). (**b**): Clinical isolate 2 (E2). (**c**): Clinical isolate 3 (E3). (**d**): Clinical isolate 4 (E4). (**e**): Clinical isolate 5 (E5). The treated disc used were AmB solution (50–200 µg/disc), AmB-SFNs (50–200 µg/disc), DMSO (0.5%) and NSS. The inhibition zone was measured from edge to edge of the clear area.

The antifungal activity of AmB-SFNs was evaluated against five clinical isolates using a disc diffusion assay and compared with conventional AmB solution (Table 2, Figure 5 and Figure 6). AmB solution demonstrated superior immediate antifungal efficacy, with maximum inhibition zones of 10.0–16.0 mm at 200 µg/disc and a consistent minimum effective concentration of 50 µg/disc across all isolates. In contrast, AmB-SFNs exhibited moderate initial activity, with maximum inhibition zones of 7.0–10.0 mm at 200 µg/disc, requiring slightly higher minimum concentrations (100 µg/disc for isolates E1–E3, 50 µg/disc for E4–E5) to achieve measurable inhibition.

The reduced immediate activity of AmB-SFNs compared to the free AmB solution is consistent with the controlled release profile from the polymer matrix system, where drug release occurs gradually rather than instantaneously [11]. This sustained release characteristic suggests potential for prolonged therapeutic activity, warranting further investigation in in vivo models to assess pharmacokinetic benefits.

To further investigate these observations, inhibition zone diameters were subjected to a two-way factorial ANOVA, with isolate (E1–E5) and concentration (50, 100, 150, 200 µg/disc) as the two factors, analyzed separately for AmB solution and AmB-SFNs (Table 3). The results demonstrated that concentration had a highly significant main effect (*p* < 0.001) for both formulations, confirming a concentration-dependent increase in antifungal efficacy. The isolate factor also showed a significant effect (*p* < 0.01), reflecting inter-isolate variability in susceptibility. Importantly, the isolate × concentration interaction was significant (*p* < 0.05), indicating that the magnitude of the concentration–response relationship differed across isolates.

**Figure 7:**
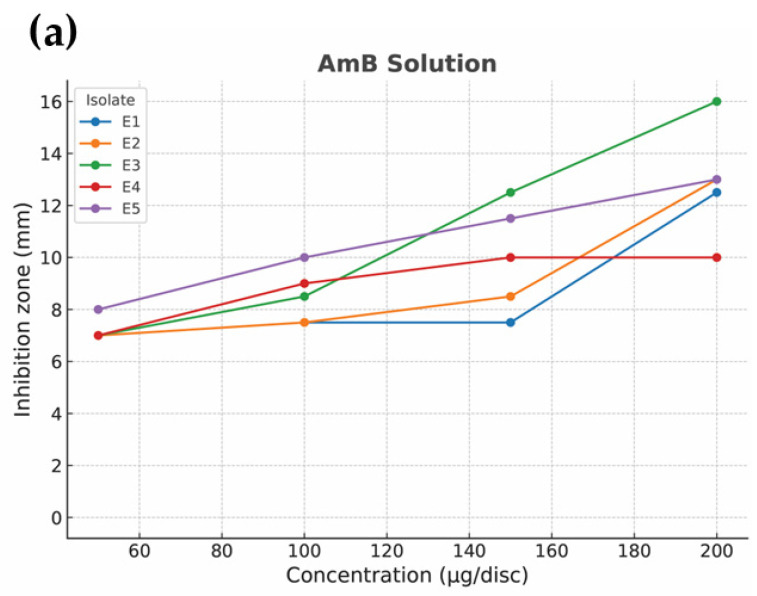

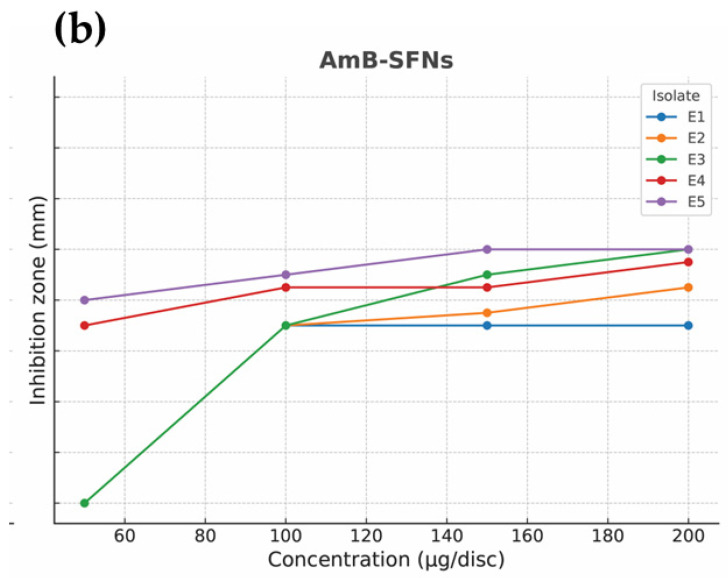
Interaction plot showing the effect of isolate and Amphotericin B concentration on inhibition zone diameters for AmB solution (**a**) and AmB-SFNs (**b**). Each line represents one clinical isolate (E1–E5), illustrating isolate-specific variations in concentration-dependent antifungal activity. For both formulations, inhibition zones increased with concentration, while differences among isolates highlight variable susceptibility. AmB-SFNs exhibited smaller inhibition zones at low concentrations but approached the activity of the solution form at higher concentrations (≥150 µg/disc), consistent with a controlled release effect.

Post hoc multiple comparisons using Tukey’s HSD revealed that isolates E3 and E5 consistently exhibited greater sensitivity across most concentrations, whereas E1 and E2 showed more moderate responses. For AmB-SFNs, inhibition zones were generally smaller at low concentrations but approached the activity of the solution form at higher concentrations (≥150 µg/disc), consistent with a controlled release effect. Figure 7 illustrates the isolate × concentration interaction plot, showing the parallel concentration-dependent trends for both formulations while highlighting isolate-specific variations in antifungal susceptibility.

Overall, these findings confirm that AmB-SFNs maintain antifungal activity against all tested isolates, indicating successful drug loading and controlled release. The observed heterogeneity among isolates aligns with typical patterns of fungal resistance [24,25], and the sustained efficacy of AmB-SFNs supports further investigation into their potential for enhanced corneal penetration, reduced systemic toxicity, and prolonged therapeutic effects in the treatment of fungal keratitis [24].

## 4. Conclusions

An ophthalmic formulation containing AmB-SFNs for topical delivery of AmB was successfully developed. Although the majority of testing in this study was conducted in vitro, the results indicate that AmB-SFNs have strong potential for suppressing corneal fungal infections, particularly those caused by *Fusarium solani*, a common pathogen in Fusarium keratitis. The AmB-SFN platform could be scaled up for industrial production and potentially adapted for the topical delivery of other drugs, especially antibiotics. This approach offers promising benefits for both patient outcomes and healthcare development.

## Figures and Tables

**Table 1:** The impact of various initial concentrations of AmB on physiochemical properties and drug entrapment efficacy of AmB-SFNs (when fix a 1% SF:Ethanol volume ratios as 1:0.2, 1% CS: 1% SF volume ratios as 1 mg/mL and amount of PEG400 as 0.1 mL). Values are expressed as mean ± SD (*n* = 3). Statistical significance was evaluated by one-way ANOVA followed by Tukey’s HSD multiple comparison test. ** *p* < 0.01, *** *p* < 0.001. Values with different lowercase superscript letters (a, b, c, d) within the same column are significantly different at *p* < 0.05.

Formulation	Initial AmpB Concentration (mg/mL)	MS ± SD (nm)	PI ± SD	ZP ± SD (mV)	EE ± SD (%)
AmB-SFNs-1	0.04	271.0 ± 13.9 ^a^ ***	0.21 ± 0.02 ^a^ **	34.35 ± 1.18 ^a^ ***	74.45 ± 1.23 ^a^ ***
AmB-SFNs-2	0.08	263.2 ± 28.3 ^a^ ***	0.20 ± 0.02 ^a^ **	39.68 ± 0.75 ^b^ ***	50.00 ± 1.82 ^b^ ***
AmB-SFNs-3	0.16	283.8 ± 15.5 ^ab^ ***	0.21 ± 0.03 ^a^ **	41.59 ± 1.04 ^b^ ***	67.24 ± 0.71 ^c^ ***
AmB-SFNs-4	0.31	333.9 ± 25.6 ^c^ ***	0.26 ± 0.05 ^b^ **	43.11 ± 1.24 ^b^ ***	60.09 ± 1.15 ^d^ ***
AmB-SFNs-5	0.45	325.4 ± 12.7 ^bc^ ***	0.25 ± 0.04 ^b^ **	53.31 ± 0.28 ^c^ ***	53.91 ± 0.51 ^b^ ***

**Table 2:** Inhibition zone diameters (mm) of clinical isolates exposed to AmB Solution and AmB-SFNs at different concentrations, determined by the disc diffusion method (*n* = 3). Values are expressed as mean ± SD.

Isolate	Concentration (µg)	AmB Solution (mm)	AmB-SFNs (mm)
E1	50	7.0 ± 0.0	0.0 ± 0.0
	100	7.5 ± 0.7	7.0 ± 0.0
	150	7.5 ± 0.7	7.0 ± 0.0
	200	12.5 ± 2.1	7.0 ± 0.0
E2	50	7.0 ± 0.0	0.0 ± 0.0
	100	7.5 ± 0.7	7.0 ± 0.0
	150	8.5 ± 2.1	7.5 ± 0.7
	200	13.0 ± 1.4	8.5 ± 2.1
E3	50	7.0 ± 0.0	0.0 ± 0.0
	100	8.5 ± 0.7	7.0 ± 0.0
	150	12.5 ± 0.7	9.0 ± 1.4
	200	16.0 ± 0.0	10.0 ± 1.4
E4	50	7.0 ± 0.0	7.0 ± 0.0
	100	9.0 ± 0.0	8.5 ± 2.1
	150	10.0 ± 0.0	8.5 ± 2.1
	200	10.0 ± 0.0	9.5 ± 2.1
E5	50	8.0 ± 0.0	8.0 ± 0.0
	100	10.0 ± 0.0	9.0 ± 1.4
	150	11.5 ± 2.1	10.0 ± 0.0
	200	13.0 ± 4.2	10.0 ± 0.0

**Table 3:** Results of two-way ANOVA analyzing the effects of isolation and concentration on inhibition zone diameters, showing sum of squares, degrees of freedom (df), *F*-values, and *p*-values for each source of variation. All factors and their interaction showed statistically significant effects (*p <* 0.001).

Formulation	Source of Variation	Sum of Squares (SS)	df	*F*-Value	*p*-Value
AmB Solution	Isolate	43.04	4	9.17	<0.001
	Concentration	212.85	3	60.44	<0.001
	Isolate × Concentration	71.32	12	5.06	<0.001
	Residual	46.95	40		
AmB-SFNs	Isolate	129.15	4	21.77	<0.001
	Concentration	321.13	3	72.18	<0.001
	Isolate × Concentration	134.24	12	7.54	<0.001
	Residual	59.32	40		

## Data Availability

Data are available from the authors. Samples of the compounds are available from the authors.

## Abbreviations

The following abbreviations are used in this manuscript:

PEG400	Polyethylene glycol 400
SF	Silk fibroin
CS	Chitosan
DI	Deionized water
AmB	amphotericin-B
AmB-SFNs	AmB-loaded fibroin nanoparticles coated with chitosan
SEM	Scanning electron microscopy
DLS	Dynamic light scattering
EE	Entrapment efficiency
SDA	Sabouraud dextrose agar
NCBI	National Center for Biotechnology Institute
CLSI	Clinical and Laboratory Standards Institute
MS	Mean size
PI	Polydispersity index
ZP	Zeta potential
SD	Standard deviation
